# Supplementary material for: Circuit-Specific Dendritic Development in the Piriform Cortex
Source: eNeuro. 2020 Jun 17;7(3):ENEURO.0083-20.2020. doi: 10.1523/ENEURO.0083-20.2020 (PMC7307633; doi:10.1523/ENEURO.0083-20.2020)
Supplement: Supplementary Figure 1-1 — Statistical analysis of the correlation between the total basal dendritic length and the vertical position of the cells in layer 2 at four time windows: 1-2 pd, 6-8 pd, 12-14 pd and >30 pd. Download Figure 1-1, DOC file [file enu-eN-NWR-0083-20-s03.doc]

**Extended Data Figure 1-1**

Data Type of Test Comparison r P-value Significant

Figure 1C1 Spearman r Location L2 vs Basal Length P1-2 0.6161 **0.0065 ****

Figure 1C2 Spearman r Location L2 vs Basal Length P6-8 0.6793 **0.001 *****

Figure 1C3 Spearman r Location L2 vs Basal Length P12-14 0.8358 **< 0.0001 ******

Figure 1C4 Spearman r Location L2 vs Basal Length P>30 0.5442 **0.0016 ****
