# Supplementary material for: Circuit-Specific Dendritic Development in the Piriform Cortex
Source: eNeuro. 2020 Jun 17;7(3):ENEURO.0083-20.2020. doi: 10.1523/ENEURO.0083-20.2020 (PMC7307633; doi:10.1523/ENEURO.0083-20.2020)
Supplement: Supplementary Table 1-2 — Statistical analysis of the intrinsic electrical properties and morphological parameters of layer 2a and layer 2b neurons at p12-14 and > p30. Download Table 1-2, DOC file [file enu-eN-NWR-0083-20-s02.doc]

**Extended Data Table 1-2**

**Data Normal distribution Type of Test Post hoc Test Comparison** **P-value** **Significant**

Table 1: Vm Yes ANOVA Holm-Sidak 2A 12-14 vs. 2B 12-14 0.9228 ns

2A 30 vs. 2B 30 0.9228 ns

2A 12-14 vs. 2A 30 0.6544 ns

2B 12-14 vs. 2B 30 0.9228 ns

Table 1: Rin Yes ANOVA Holm-Sidak 2A 12-14 vs. 2B 12-14 0.3939 ns

2A 30 vs. 2B 30 0.3939 ns

2A 12-14 vs. 2A 30 0.3939 ns

2B 12-14 vs. 2B 30 0.3939 ns

Table 1: Tau Yes ANOVA Holm-Sidak 2A 12-14 vs. 2B 12-14 0.8834 ns

2A 30 vs. 2B 30 0.8834 ns

2A 12-14 vs. 2A 30 0.5356 ns

2B 12-14 vs. 2B 30 0.9123 ns

Table 1: Cm Yes ANOVA Holm-Sidak 2A 12-14 vs. 2B 12-14 0.14 ns

2A 30 vs. 2B 30 0.2833 ns

2A 12-14 vs. 2A 30 0.3765 ns

2B 12-14 vs. 2B 30 0.0654 ns

Table 1: Threshold Yes ANOVA Holm-Sidak 2A 12-14 vs. 2B 12-14 0.3356 ns

2A 30 vs. 2B 30 0.9743 ns

2A 12-14 vs. 2A 30 0.113 ns

2B 12-14 vs. 2B 30 0.8885 ns

Table 1: FAHP Yes ANOVA Holm-Sidak 2A 12-14 vs. 2B 12-14 0.5225 ns

2A 30 vs. 2B 30 0.3886 ns

2A 12-14 vs. 2A 30 0.3813 ns

2B 12-14 vs. 2B 30 0.5225 ns

Table 1: Instan Firing Freq No Kruskal-Wallis Dunn 2A 12-14 vs. 2B 12-14 > 0.9999 ns

2A 30 vs. 2B 30 > 0.9999 ns

2A 12-14 vs. 2A 30 > 0.9999 ns

2B 12-14 vs. 2B 30 > 0.9999 ns

Figure 2A2: Apical N branches Yes ANOVA Holm-Sidak 2A 1-2 vs. 2A 6-8 0.3652 ns

2A 6-8 vs. 2A 12-14 0.9212 ns

2A 12-14 vs. 2A 30 0.8258 ns

2B 1-2 vs. 2B 6-8 0.0004 *******

2B 6-8 vs. 2B 12-14 0.6565 ns

2B 12-14 vs. 2B 30 0.0401 *****

2A 1-2 vs. 2B 1-2 0.4625 ns

2A 6-8 vs. 2B 6-8 0.4625 ns

2A 12-14 vs. 2B 12-14 0.4625 ns

2A 30 vs. 2B 30 0.1986 ns

Figure 2A3: Apical dendritic length Yes ANOVA Holm-Sidak 2A 1-2 vs. 2A 6-8 0.4547 ns

2A 6-8 vs. 2A 12-14 0.0237 *****

2A 12-14 vs. 2A 30 0.0151 *****

2B 1-2 vs. 2B 6-8 0.0215 *****

2B 6-8 vs. 2B 12-14 0.0078 ******

2B 12-14 vs. 2B 30 0.3651 ns

2A 1-2 vs. 2B 1-2 0.8141 ns

2A 6-8 vs. 2B 6-8 0.4914 ns

2A 12-14 vs. 2B 12-14 0.4914 ns

2A 30 vs. 2B 30 0.0321 *****

Figure 2A4: Apical N stems Yes ANOVA Holm-Sidak 2A 1-2 vs. 2A 6-8 0.0063 ******

2A 6-8 vs. 2A 12-14 0.9102 ns

2A 12-14 vs. 2A 30 0.0063 ******

2B 1-2 vs. 2B 6-8 0.4802 ns

2B 6-8 vs. 2B 12-14 0.4802 ns

2B 12-14 vs. 2B 30 0.5603 ns

2A 1-2 vs. 2B 1-2 0.1932 ns

2A 6-8 vs. 2B 6-8 0.0004 *******

2A 12-14 vs. 2B 12-14 < 0.0001 ********

2A 30 vs. 2B 30 0.0996 ns

Figure 2B: Avg Apical dendritic length Yes ANOVA Holm-Sidak 2A 1-2 vs. 2A 6-8 0.3912 ns

2A 6-8 vs. 2A 12-14 < 0.0001 ****

2A 12-14 vs. 2A 30 < 0.0001 ********

2B 1-2 vs. 2B 6-8 0.116 ns

2B 6-8 vs. 2B 12-14 0.0004 *******

2B 12-14 vs. 2B 30 0.0229 *****

2A 1-2 vs. 2B 1-2 0.779 ns

2A 6-8 vs. 2B 6-8 0.5553 ns

2A 12-14 vs. 2B 12-14 0.779 ns

2A 30 vs. 2B 30 0.5553 ns

Figure 3A2: Basal N branches Yes ANOVA Holm-Sidak 2A 1-2 vs. 2A 6-8 0.3469 ns

2A 6-8 vs. 2A 12-14 0.8511 ns

2A 12-14 vs. 2A 30 0.588 ns

2A 1-2 vs. 2A 30 0.0478 *****

2B 1-2 vs. 2B 6-8 0.0066 ******

2B 6-8 vs. 2B 12-14 0.0949 ns

2B 12-14 vs. 2B 30 0.0299 *****

2A 1-2 vs. 2B 1-2 0.0587 ns

2A 6-8 vs. 2B 6-8 0.0002 *******

2A 12-14 vs. 2B 12-14 < 0.0001 ********

2A 30 vs. 2B 30 0.0055 ******

Figure 3A3: Basal dendritic length Yes ANOVA Holm-Sidak 2A 1-2 vs. 2A 6-8 0.4705 ns

2A 6-8 vs. 2A 12-14 0.2269 ns

2A 12-14 vs. 2A 30 0.2269 ns

2A 1-2 vs. 2A 30 0.0017 ******

2B 1-2 vs. 2B 6-8 0.0115 *****

2B 6-8 vs. 2B 12-14 0.0002 *******

2B 12-14 vs. 2B 30 0.0018 ******

2A 1-2 vs. 2B 1-2 0.2701 ns

2A 6-8 vs. 2B 6-8 0.0017 ******

2A 12-14 vs. 2B 12-14 < 0.0001 ********

2A 30 vs. 2B 30 0.0151 *****

Figure 3A4: Basal N stems Yes ANOVA Holm-Sidak 2A 1-2 vs. 2A 6-8 0.9587 ns

2A 6-8 vs. 2A 12-14 0.8718 ns

2A 12-14 vs. 2A 30 0.9587 ns

2B 1-2 vs. 2B 6-8 0.9613 ns

2B 6-8 vs. 2B 12-14 0.0983 ns

2B 12-14 vs. 2B 30 0.0037 ******

2A 1-2 vs. 2B 1-2 0.009 ******

2A 6-8 vs. 2B 6-8 0.0048 ******

2A 12-14 vs. 2B 12-14 < 0.0001 ********

2A 30 vs. 2B 30 0.1137 ns

Figure 3B: Avg Basal dendritic length Yes ANOVA Holm-Sidak 2A 1-2 vs. 2A 6-8 0.7039 ns

2A 6-8 vs. 2A 12-14 0.1208 ns

2A 12-14 vs. 2A 30 0.2627 ns

2A 1-2 vs. 2A 30 0.0034 ******

2B 1-2 vs. 2B 6-8 0.421 ns

2B 6-8 vs. 2B 12-14 < 0.0001 ********

2B 12-14 vs. 2B 30 0.2488 ns

2A 1-2 vs. 2B 1-2 0.9307 ns

2A 6-8 vs. 2B 6-8 0.9307 ns

2A 12-14 vs. 2B 12-14 0.3307 ns

2A 30 vs. 2B 30 0.5864 ns

Figure 4D: Avg % coactive neurons Yes Unpaired t-test 1/3 L2 vs 3/3 L2 0.001801 ******

Figure 5B1: Apical N branches in L1A Yes ANOVA Holm-Sidak 2A 1-2 vs. 2A 6-8 0.0093 ******

2A 6-8 vs. 2A 12-14 0.2966 ns

2A 12-14 vs. 2A 30 0.2116 ns

2B 1-2 vs. 2B 6-8 < 0.0001 ********

2B 6-8 vs. 2B 12-14 0.6689 ns

2B 12-14 vs. 2B 30 0.0218 *****

2A 1-2 vs. 2B 1-2 0.4472 ns

2A 6-8 vs. 2B 6-8 0.4472 ns

2A 12-14 vs. 2B 12-14 0.4472 ns

2A 30 vs. 2B 30 0.0038 ******

Figure 5B2: Apical N int branches in L1A Yes ANOVA Holm-Sidak 2A 1-2 vs. 2A 6-8 0.0066 ******

2A 6-8 vs. 2A 12-14 0.0606 ns

2A 12-14 vs. 2A 30 0.0606 ns

2B 1-2 vs. 2B 6-8 < 0.0001 ********

2B 6-8 vs. 2B 12-14 0.2506 ns

2B 12-14 vs. 2B 30 0.0337 *****

2A 1-2 vs. 2B 1-2 0.4658 ns

2A 6-8 vs. 2B 6-8 0.4658 ns

2A 12-14 vs. 2B 12-14 0.3362 ns

2A 30 vs. 2B 30 0.0033 ******

Figure 5B3: Apical N tips branches in L1A Yes ANOVA Holm-Sidak 2A 1-2 vs. 2A 6-8 0.0258 *****

2A 6-8 vs. 2A 12-14 0.8365 ns

2A 12-14 vs. 2A 30 0.522 ns

2B 1-2 vs. 2B 6-8 < 0.0001 ********

2B 6-8 vs. 2B 12-14 0.9079 ns

2B 12-14 vs. 2B 30 0.0222 *****

2A 1-2 vs. 2B 1-2 0.3281 ns

2A 6-8 vs. 2B 6-8 0.4732 ns

2A 12-14 vs. 2B 12-14 0.4732 ns

2A 30 vs. 2B 30 0.0088 ******

Figure 5C1: Apical dendritic length in L1A Yes ANOVA Holm-Sidak 2A 1-2 vs. 2A 6-8 0.2541 ns

2A 6-8 vs. 2A 12-14 0.084 ns

2A 12-14 vs. 2A 30 0.0009 *******

2B 1-2 vs. 2B 6-8 0.0149 *****

2B 6-8 vs. 2B 12-14 0.0644 ns

2B 12-14 vs. 2B 30 0.3158 ns

2A 1-2 vs. 2B 1-2 0.7608 ns

2A 6-8 vs. 2B 6-8 0.7295 ns

2A 12-14 vs. 2B 12-14 0.7295 ns

2A 30 vs. 2B 30 0.0002 *******

Figure 5C1: Avg Apical dendritic length in L1A Yes ANOVA Holm-Sidak 2A 1-2 vs. 2A 6-8 0.5613 ns

2A 6-8 vs. 2A 12-14 < 0.0001 ********

2A 12-14 vs. 2A 30 0.0031 ******

2B 1-2 vs. 2B 6-8 0.1166 ns

2B 6-8 vs. 2B 12-14 0.0351 *****

2B 12-14 vs. 2B 30 0.0002 *******

2A 1-2 vs. 2B 1-2 0.9215 ns

2A 6-8 vs. 2B 6-8 0.8023 ns

2A 12-14 vs. 2B 12-14 0.9215 ns

2A 30 vs. 2B 30 0.0142 *****

Figure 6C1: AUC ACSF vs. APV Yes (but n=5) Paired t-test L2A ACSF vs L2A APV 0.294 ns

L2B ACSF vs L2B APV 0.0126 *****

Wilcoxon L2A ACSF vs L2A APV 0.686 ns

L2B ACSF vs L2B APV 0.043 *****

Figure 6C1: AUC L2A APV vs. L2B APV Yes (but n=5) Unpaired t-test L2A APV vs L2B APV 0.924 ns

Mann-Whitney L2A APV vs L2B APV 1 ns

Figure 6C2: Amp ACSF vs. APV Yes (but n=5) Paired t-test L2A ACSF vs L2A APV 0.891 ns

L2B ACSF vs L2B APV 0.0117 *****

Wilcoxon L2A ACSF vs L2A APV 0.893 ns

L2B ACSF vs L2B APV 0.043 *****

Figure 6C2: Amp L2A APV vs. L2B APV Yes (but n=5) Unpaired t-test L2A APV vs L2B APV 0.248 ns

Mann-Whitney L2A APV vs L2B APV 0.296 ns

Figure 6D: ∆G/R ACSF vs. APV Yes (but n=5) Ratio paired t-test L2A ACSF vs L2A APV 0.0187 *****

L2B ACSF vs L2B APV 0.0025 ******

Wilcoxon test L2A ACSF vs L2A APV 0.043 *****

L2B ACSF vs L2B APV 0.043 *****

Figure S2B: Apical N tips branches in L2 Yes ANOVA Holm-Sidak 2A 1-2 vs. 2A 6-8 0.5659 ns

2A 6-8 vs. 2A 12-14 0.8522 ns

2A 12-14 vs. 2A 30 0.8522 ns

2B 1-2 vs. 2B 6-8 0.4691 ns

2B 6-8 vs. 2B 12-14 0.9603 ns

2B 12-14 vs. 2B 30 0.9603 ns

Figure S2C: Apical N tips branches in L1B No Kruskal-Wallis Dunn 2 A 1-2 vs. 2A 6-8 0.0459 *****

2A 6-8 vs. 2A 12-14 0.2791 ns

2A 12-14 vs. 2A 30 > 0.9999 ns

2B 1-2 vs. 2B 6-8 > 0.9999 ns

2B 6-8 vs. 2B 12-14 0.0078 ******

2B 12-14 vs. 2B 30 0.263 ns

2A 1-2 vs. 2B 1-2 0.003 ******

2A 6-8 vs. 2B 6-8 > 0.9999 ns

2A 12-14 vs. 2B 12-14 > 0.9999 ns

2A 30 vs. 2B 30 0.1112 ns
